# A New Functional Food Ingredient Obtained from *Aloe ferox* by Spray Drying

**DOI:** 10.3390/foods12040850

**Published:** 2023-02-16

**Authors:** Francesca Comas-Serra, Juan José Martínez-García, Alma Pérez-Alba, María de los Ángeles Sáenz-Esqueda, María Guadalupe Candelas-Cadillo, Antoni Femenia, Rafael Minjares-Fuentes

**Affiliations:** 1 Department of Chemistry, University of the Balearic Islands, Ctra Valldemossa Km 7.5, 07122 Palma de Mallorca, Spain; 2 Facultad de Ciencias Químicas, Universidad Juárez del Estado de Durango, Av. Artículo 123 s/n Fracc, Filadelfia, Gómez Palacio 35010, Durango, Mexico

**Keywords:** *Aloe ferox*, spray drying, acetylated mannan, ^1^H NMR, antioxidant capacity

## Abstract

Aloe mucilages of *Aloe ferox* (*A. ferox*) and *Aloe vera* (*A. vera*) were spray-dried (SD) at 150, 160 and 170 °C. Polysaccharide composition, total phenolic compounds (TPC), antioxidant capacity and functional properties (FP) were determined. *A. ferox* polysaccharides were comprised mainly of mannose, accounting for >70% of SD aloe mucilages; similar results were observed for A. *vera*. Further, an acetylated mannan with a degree of acetylation >90% was detected in *A. ferox* by ^1^H NMR and FTIR. SD increased the TPC as well as the antioxidant capacity of *A. ferox* measured by both ABTS and DPPH methods, in particular by ~30%, ~28% and ~35%, respectively, whereas in *A. vera*, the antioxidant capacity measured by ABTS was reduced (>20%) as a consequence of SD. Further, FP, such as swelling, increased around 25% when *A. ferox* was spray-dried at 160 °C, while water retention and fat adsorption capacities exhibited lower values when the drying temperature increased. The occurrence of an acetylated mannan with a high degree of acetylation, together with the enhanced antioxidant capacity, suggests that SD *A. ferox* could be a valuable alternative raw material for the development of new functional food ingredients based on Aloe plants.

## 1. Introduction

In recent decades, the Aloe genus has been studied widely with the aim of obtaining new sources of gums and hydrocolloids. This is mainly due to the high demand for functional ingredients able to replace, either partially or totally, synthetic molecules considered potentially harmful to human health [1,2]. Within this context, *Aloe vera* has been the most exploited variety, mainly due to its wide gamma of associated beneficial properties, which have been attributed to the presence of the main bioactive polysaccharide acemannan, an acetylated mannose-rich polysaccharide [3,4,5,6,7] considered as a storage polysaccharide from *Aloe vera* gel and it is located within the protoplast of the parenchymatous cells [4].

*Aloe vera* mucilage has been widely used for the development of new functional food ingredients, the microencapsulation of bioactive compounds by spray drying being one of the most studied applications since *Aloe vera* mucilage is considered a biodegradable material and generally safer than other polymers [8,9,10]. However, it has been reported that spray drying of *Aloe vera* gel performed on an industrial scale might lead to the degradation of the bioactive acemannan polymer, with the loss of acetyl groups (deacetylation) and the reduction of its molecular weight being the structural features mostly affected [5,11]. This may lead to the loss of important biological and functional properties associated with *Aloe vera* gel [3,5,12].

*Aloe ferox*, commonly known as the bitter aloe or Cape aloe, has been used in traditional medicine since ancient times and is probably the most used variety following *Aloe vera*. In fact, in 2002, the Food and Drug Administration approved its use as a direct food additive for human consumption as a natural flavoring substance [13]. In Africa and Europe, *Aloe ferox* gel has been used as a laxative medicine and is considered to have bitter tonic, antioxidant, anti-inflammatory, antimicrobial and anticancer properties [14]. Nevertheless, the chemical composition of *Aloe ferox* polysaccharides remains unclear since, initially, pectic polysaccharides, specifically arabinogalactans, were considered as its predominant polymers, followed by glucomannans [15]. Later, other studies showed that the predominant polymers were mainly composed of glucose and galactose, suggesting galactose as a possible fingerprint for *Aloe ferox* [16]. Thus, these differences in the compositional features of *Aloe ferox* polysaccharides could be an advantage in preserving the biological activity during the obtention of functional food ingredients, in particular when spray drying is applied. However, to the best of our knowledge, there is no information about the compositional changes of *Aloe ferox* polysaccharides and their related functional properties when the mucilage is subjected to a spray drying procedure. Therefore, the main aim of this study was to assess the changes in the polysaccharide composition of spray-dried *Aloe ferox* gel and its related functional properties, comparing it with spray-dried *Aloe vera*. It is important to highlight that the results of this study could be used not only for the development of new functional food ingredients but also to update the available scientific information about the composition of *Aloe ferox* polysaccharides.

## 2. Materials and Methods

### 2.1. Raw Material

All aloe leaves of both *Aloe ferox* (*A. ferox*) and *Aloe vera* (*A. vera*) were obtained from the experimental field of the “Facultad de Agricultura y Zootecnia (FAZ)” which belongs to the Universidad Juárez del Estado de Durango (Venecia, Durango, Mexico). All the aloe leaves were obtained from 3-year-old plants. The filets from each Aloe variety were manually extracted as previously described in Rodríguez–González et al. [6]. The mucilages from both Aloe varieties were obtained using a commercial juice extractor Health-Smart (Hamilton Beach^®^, Mexico, Mexico). Finally, they were filtered using a nylon cloth in order to remove any fibrous material.

### 2.2. Spray Drying (SD)

*Aloe ferox* mucilage was dehydrated by spray-drying (SD) using a Laboratory Spray dryer SD Basic (LabPlant, North Yorkshire, UK). Approximately 500 g of *A. ferox* were dehydrated using constant feed flow (0.4 L/h). The mucilage was dehydrated at three different inlet temperatures: 150, 160, and 170 °C, and the outlet temperatures were 88, 94, and 100 °C, respectively. One sample of fresh *A. ferox* mucilage was freeze-dried (FD) in a FreeZone 6 Benchtop Lyophilizer (Labconco, Kansas City, MO, USA) operating at −40 °C and 0.07 mBar and taken as a reference treatment. Furthermore, *Aloe vera* (*A. vera*) mucilage was processed (SD and FD) under the same conditions for comparison purposes [5].

All the dehydrated Aloe samples were packed in polyethylene bags, hermetically sealed and stored in a desiccator with silica gel in order to avoid absorption of moisture [5].

### 2.3. Scanning Electron Microscopy (SEM)

The morphology of both Aloe mucilages, *A. ferox* and *A. vera*, dehydrated by SD, was observed using an SEM S-3400N (Hitachi, Tokyo, Japan). Micrographs were taken with an accelerating voltage of 15 kV. Samples were coated with gold and observed directly at 40 Pa of pressure.

### 2.4. Color

The color characteristics of all dehydrated Aloe samples were determined using a spectrophotometer CM-5 (Konica Minolta, Tokyo, Japan) as previously described by Minjares–Fuentes et al. [5]. Chroma parameter (*C*), indicating color intensity, and hue angle (*H°*) were calculated using Equations (1) and (2), respectively.

(1) C=(a*2+b*2)12

(2) $$H{}^{\circ}=tan^{-1}\left(\frac{b^{*}}{a^{*}}\right)$$

Total color difference (Δ*E*) was also calculated using Equation (3):

(3) $$\Delta E=\sqrt{{\left(L_{0}^{*}-L^{*}\right)}^{2}+{\left(a_{0}^{*}-a^{*}\right)}^{2}+{\left(b_{0}^{*}-b^{*}\right)}^{2}}$$

where $L_{0}^{*}$, $a_{0}^{*}$ and $b_{0}^{*}$ are the values of the FD sample, and $L^{*}$, $a^{*}$ and $b^{*}$ the measured values corresponding to a SD sample. All parameters were measured at least in triplicate for each treatment.

### 2.5. Total Phenolic Content (TPC) and Antioxidant Capacity

The TPC of dehydrated aloe samples was determined using the Folin-Ciocalteu method, as previously described by Medina–Torres et al. [17]. TPC was calculated using a calibration curve of gallic acid (Sigma-Aldrich, Mexico City, Mexico) content ranging from 10 to 120 μg/mL and expressed as mg of gallic acid per 100 g of dry matter (mg GA/100 g dm). The effect of SD processing on the antioxidant capacity of Aloe samples was assessed by ABTS (2,2-Azino-bis(3-ethylbenzthiazoline-6-sulfonic acid)) (Sigma-Aldrich, Mexico City, Mexico) assay as previously described by González–Centeno et al. [18], while the DPPH (2,2-diphenyl-1-picrylhydrazyl; Sigma–Aldrich, Mexico City, Mexico) free radical scavenging capacity was measured following the procedure described in Medina–Torres et al. [19].

### 2.6. Polysaccharide Analysis

#### 2.6.1. Alcohol Insoluble Residues (AIRs) Preparation

AIRs from dehydrated Aloe samples were obtained by immersion in boiling ethanol as described in Femenia et al. [20] and sieved through a ∅ 0.5 mm mesh.

#### 2.6.2. Carbohydrate Analysis

Carbohydrate analysis was performed as described by Alvarado–Morales et al. [21] for neutral sugars. Sugars were released from residues by acid hydrolysis. Neutral sugars were derivatized as their alditol acetates and isothermally separated at 220 °C by GC (Hewlett Packard 5890A, Waldbronn, Germany) with an FID detector and equipped with a 30 m column DB-225 (J&W Scientific, Folsom, CA, USA) with i.d. and film thickness of 0.25 mm and 0.15 μm, respectively. Uronic acids were determined as total uronic acid by colorimetry according to the methodology proposed by Blumenkrantz and Asboe–Hansen [22].

### 2.7. FTIR and ^1^H NMR Analysis

FTIR spectra of Aloe dehydrated samples were obtained using a Bruker Tensor 27 FTIR spectrometer (Bruker, Billerica, MA, USA), and ^1^H NMR analysis was performed in a Bruker Avance 300 spectrometer (Bruker, Billerica, MA, USA) at 300.13 MHz as previously described by Minjares–Fuentes et al. [2].

### 2.8. Functional Properties

Swelling (Sw) and water retention capacity (WRC), the main hydration properties, as well as fat adsorption capacity (FAC) were the functional properties determined in this study. Thus, the Sw and WRC of AIRs from aloe-dehydrated samples were measured in phosphate buffer (1 M; pH 6.3), and the FAC was measured using sunflower oil. These techno-functional properties were determined following the methodology described by Alvarado–Morales et al. [21].

### 2.9. Statistical Analysis

The effects of the SD procedure on the color, TPC, antioxidant capacity, carbohydrate composition, and functional properties of both dehydrated Aloe mucilages, *A. ferox* and *A. vera*, were evaluated using one-way analysis of variance (ANOVA) with a significance level α = 0.05. Further, the Fisher LSD test was used for post hoc analysis. All calculations were performed using Minitab software (Minitab version 19, Penn State University, PA, USA).

## 3. Results and Discussion

### 3.1. SEM Observations

**Figure 1:**
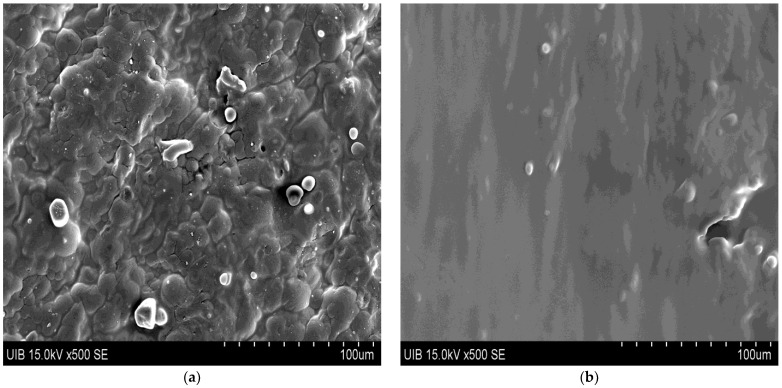

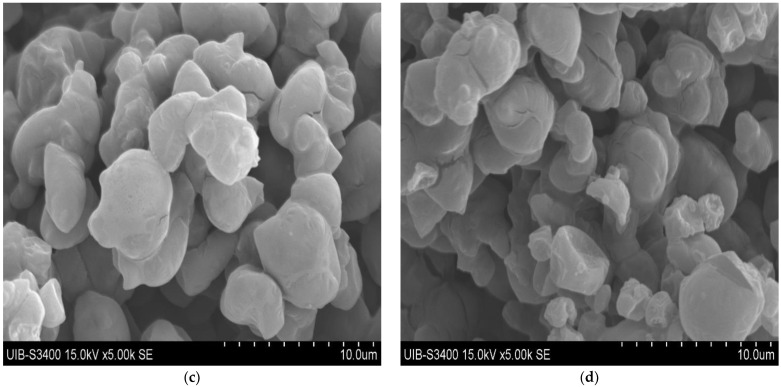
Micrographs of *A. ferox* (**a**,**c**) and *A. vera* (**b**,**d**) mucilages dehydrated by FD and SD, respectively.

SEM micrographs corresponding to *A. ferox* and *A. vera* samples obtained by FD and SD are shown in Figure 1. As can be seen, the FD aloe samples, either *A. ferox* (Figure 1a) or *A. vera* (Figure 1b), showed a laminar shape and rough surface with some structural cracks, which have also been observed by other authors [10,19]. On the contrary, all the SD aloe samples, both of *A. ferox* (Figure 1c) and *A. vera* (Figure 1d), presented a spherical and/or oval shape and smooth surface particles. Interestingly, this type of morphology has also been described in the literature for other products dehydrated by SD, such as mucilages [19,23] and fruit juices [24,25], among others. It is important to note that the morphological features obtained after applying the spray drying process suggest the potential use of SD *A. ferox* mucilage as a microencapsulating agent of different bioactive compounds [8,9,10].

### 3.2. Color

Color constitutes an important tool for evaluating the overall quality of Aloe products since color changes caused by different degradation processes can usually be observed when Aloe plants are being treated [26,27].

Thus, the color of the dehydrated Aloe mucilage for both *A. ferox* and *A. vera* was measured through the CIEL a* b* color coordinates and the results are summarized in Table 1. As can be seen, FD *A. ferox* (*L* = 74.1) was duller than FD *A. vera* (*L* = 87.4) (*p* < 0.05), whereas no significant differences were observed in the a*, b*, *C* and *H*° parameters of both samples (*p* > 0.05). Interestingly, SD led to a reduction in the lightness (*L*) of all the SD aloe mucilages, both *A. ferox* and *A. vera*, as the drying temperature increased from 150 °C to 170 °C, reducing its value from 85.7 to 77.9 in SD *A. ferox* and from 83.1 to 77.6 in the case of SD *A. vera*. On the other hand, the a*, b* and *C* parameters of both SD aloe mucilages increased while *H*° decreased as SD temperature increased (see Table 1). Thus, for SD *A. ferox*, a* and b* parameters increased from 1.4 to 3.1 and from 17.0 to 19.3, respectively, whereas for SD *A. vera*, these parameters increased from 1.8 to 4.2 and from 16.0 to 19.7, respectively. Further, *C* increased from 17.1 to 19.6 in SD *A. ferox* and from 16.1 to 20.2 in SD *A. vera* when SD temperature increased. Finally, with regard to the *H*°, the increase in SD temperature caused the reduction of this parameter, from 85.3 to 81.0 for SD A. *ferox* and from 83.4 to 77.9 for *A. vera* (*p* < 0.05).

The Δ*E* is probably one of the most important color parameters, as it enables us to quantify the overall color modification of a sample caused by processing in comparison with a reference sample [21]. In this study, FD samples of both *A. ferox* and *A. vera* were used as references for each variety. Interestingly, the Δ*E* for *A. ferox* decreased from 11.7 to 5.4, as the drying temperature increased from 150 to 170 °C, whereas that of *A. vera* increased from 5.8 up to 11.3. Given these results, clearly, SD caused color changes higher than 2 units which can be observed by the naked eye [28].

Furthermore, the better preservation of the color characteristics in *A. ferox*, associated with the low Δ*E* and high L values, could be attributed to a minor degradation of the different types of bioactive compounds since it has been observed that SD could lead to thermal and oxidative degradation of phenolic compounds and free sugars, producing either off-colors or browning compounds [29,30]. Therefore, the color characteristics of SD *A. ferox* mucilage could be an important physicochemical parameter to be taken into account in the selection of raw materials used for the development of new products not only in the agro-food sector but also in the pharmaceutical and cosmetic fields.

### 3.3. TPC and Antioxidant Capacity

**Figure 2:**
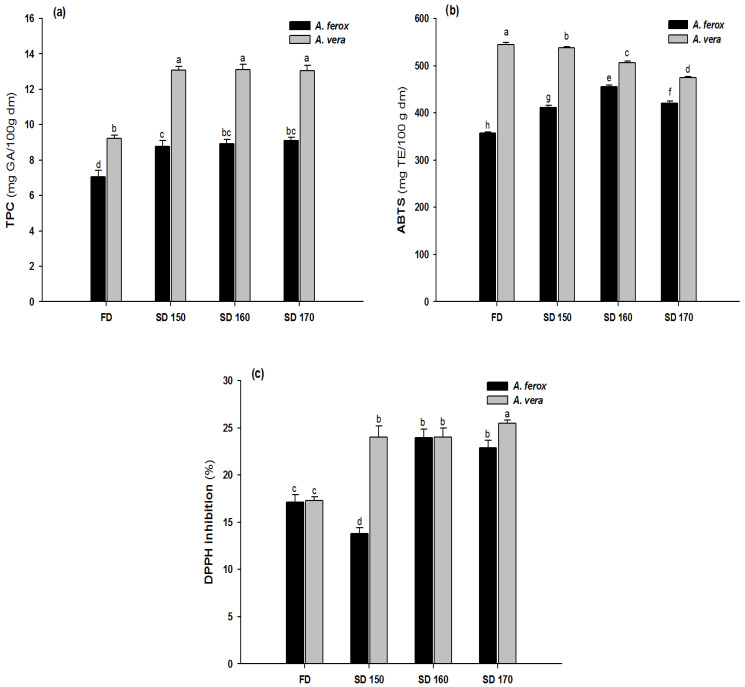
TPC (**a**), ABTS+ (**b**) and DPPH (**c**) of *A. ferox* and *A. vera* dehydrated by FD and SD at different temperatures. Lowercase letters (a, b, c, d, e, f, g, h) above the bars indicate statistical difference in samples (*p* < 0.05).

The results from the TPC present in the Aloe samples analyzed are shown in Figure 2. As can be seen, in general, the TPC values were significantly lower in *A. ferox* than those measured for *A. vera* (*p* < 0.05). In particular, the TPC of *A. ferox* ranged from 7.1 to 9.0 mg GA/100g dm, whereas in *A. vera*, they ranged from 9.2 to 13.1 mg GA/100g dm (Figure 2a). Interestingly, the SD procedure led to a significant increase of TPC in both dehydrated mucilages (*p* < 0.05). So, in comparison with the FD mucilages, the TPC from *A. ferox* showed an increase of around 30% as a consequence of SD, whereas an increase higher than 35% was observed in the case of SD *A. vera*. These results disagree with those reported by Medina–Torres et al. [19], who observed some loss of phenolic compounds of *Aloe vera* mucilage dehydrated by SD. The degradation of bioactive compounds during the SD process has been attributed, on the one hand, to long residence times due to low feed-flows (<1.5 L/h) and, on the other hand, to the high shear forces which appear in the drying chamber, by the combination of high feed-flow, low atomization pressure and high drying temperatures [19]. Nevertheless, it has also been observed that Aloe polysaccharides could be able to link to other bioactive compounds, including phenolic compounds, encapsulating those and forming a resistant structure which could explain the high retention of the phenolic content observed in this study [8,10].

The antioxidant capacity of *A. ferox*, measured by the ABTS assay, was lower than for *A. vera* (*p* < 0.05) (see Figure 2b). However, the SD procedure caused a significant increase in its antioxidant capacity (ABTS assay), increasing from ~350 mg TE/100 g dm in the FD sample up to ~450 mg TE/100 g dm in SD 160 (*p* < 0.05). On the contrary, for the *A. vera* mucilage, the antioxidant capacity, measured by the ABTS method, decreased from ~550 up to ~450 mg TE/100 g dm (*p* < 0.05) as the drying temperature increased from 150 to 170 °C (see Figure 2b). The reduction of the antioxidant capacity has mainly been attributed to the thermal degradation of different bioactive compounds, including phenolic compounds [19]. Interestingly, the antioxidant capacity of Aloe species has recently been associated with the presence of two main bioactive compounds, aloeresin A and coumaroylaloesin, with the latter being only found in *A. ferox* [31]. Thus, the loss of antioxidant capacity of *A. vera* could be due to the degradation of aloeresin A as a consequence of the increase in the drying temperature.

With regard to the DPPH assay, the radical scavenging activity increased from ~17%, in both FD samples, up to ~23% and ~25% for *A. ferox* SD 160 and *A. vera* SD 170, respectively (see Figure 2c). It has been observed that the application of adequate SD conditions could cause high retention of phenolic compounds, as well as an increase in antioxidant capacity. This could be attributed to the short residence times (<5 s) in the drying chamber as a consequence of the high feed flows (1.5 L/h) used at low SD temperature (150 °C) [19].

Mucilages have widely been used for the microencapsulation of different bioactive compounds such as gallic acid [8,32], curcumin [9], and carotenoids [10], among others [33,34]. Within this context, it has been observed that *A. vera* mucilage offers better retention of bioactive compounds than other mucilages during microencapsulation by the SD procedure [8,9,10]. In fact, it has been reported that gallic acid microencapsulated using *A. vera* shows higher EC_50_ (~540 mg) than when microencapsulation is carried out using maltodextrin (EC_50_ = ~590 mg) [8]. The enhancement of the antioxidant capacity has been attributed to the interaction between the encapsulated compound, such as gallic acid, and the bioactive compounds present in the *A. vera* mucilage [10].

Overall, it can be inferred that *A. ferox* could be an excellent alternative source for the microencapsulation process of different bioactive compounds since the antioxidant properties were well preserved during the SD procedure, as can be seen from the results obtained in the ABTS assay.

### 3.4. Polysaccharide Composition

Aloe plants are highly valorized by their polysaccharide composition since most of their functional and biological properties have been associated with these biocompounds [35]. Therefore, all dehydrated Aloe samples were subjected to carbohydrate analysis in order to obtain information about their polysaccharide composition. The GC analysis revealed the presence of high amounts of mannose and uronic acids (UA), followed by glucose and galactose. Also, to a minor extent, rhamnose and arabinose were detected in all samples, while small amounts of fucose and xylose were mainly quantified in the FD samples (see Table 2).

Interestingly, the mannose content was similar in most of the SD samples (>72%) for both *A. ferox* and *A. vera*, whereas in the FD samples, mannose accounted for ~60% of *A. ferox* and ~70% of *A. vera*. It is important to highlight that the presence of large amounts of mannose in *Aloe vera* has been associated with the occurrence of the bioactive polymer acemannan [3,4,5,36]. On the other hand, the glucose content was significantly higher in the FD samples, accounting for ~21% in *A. ferox* and ~15% in *A. vera*, whereas in the SD aloe mucilages, it was lower than 10%. Interestingly, SD *A. ferox* showed a lower glucose content than SD *A. vera*. Specifically, glucose varied from 7.5 to 8.0% in SD A. *ferox*, whereas in SD *A. vera*, it ranged from 8.7% to 10.3%. Probably, the considerable glucose reduction, together with the loss of xylose, observed after SD could be the result of thermal and mechanical degradation of xyloglucans present in both aloe mucilages. It is also important to note that mannose was better preserved than glucose after the SD procedure, suggesting minor variations in the molecular structure of this mannose-rich polymer [19].

However, *A. ferox* showed significantly higher amounts of UA than *A. vera*. It varied from 10.0% up to 16.9% in *A. ferox* samples, whereas in *A. vera*, it ranged from 7.9% to 9.2%. The presence of large amounts of UA has been associated with the occurrence of pectic polysaccharides, such as homogalacturonans, which are considered the main pectic-polymer type present in *A. vera* [36,37,38].

Overall, these results disagree with those reported by Mabusela et al. [15] and O’Brien et al. [16], who analyzed the polysaccharide composition of *A. ferox* leaf gel. Previously, pectic polysaccharides were found to be the predominant polymer present in the water-soluble fractions of *A. ferox*, while glucomannans were mainly found in alkali extracts [15], this being an indication of the presence of hemicellulosic polysaccharides released from cell walls. However, later, O’Brien et al. [16] observed that *A. ferox* leaf gel was mainly composed of high amounts of glucose, followed by galactose and minor amounts of xylose and arabinose. These authors pointed out the presence of galactose as the characteristic monomer in *A. ferox*, suggesting this as a possible useful fingerprint for *A. ferox*, as mannose is for *A. vera*. It is important to highlight that O’Brien et al. [16] did not identify mannose residues after hydrolysis of *A. ferox* gel, which disagrees with the findings of Mabusela et al. [15] and also with the results obtained in this study.

Although there are few studies based on the polysaccharide composition of *A. ferox* gel, some of the observed variations could be attributed not only to the geographical location but also to the agricultural management of the Aloe plants since it has been observed that these factors may lead to important compositional and structural variations in the Aloe polysaccharides [2,36].

It is also worth mentioning the small but significant differences observed in the polysaccharide composition of *A. ferox*, specifically for pectic polysaccharides, together with the presence of considerable amounts of a mannose-rich polymer, similar to the acemannan polymer. This fact could be a technological advantage for the development of functional food products from *A. ferox*.

### 3.5. FTIR and ^1^H NMR Analysis of Aloe Samples

FTIR and ^1^H NMR analyses have been considered useful tools for the characterization of Aloe powders [3,36,39,40].

**Figure 3:**
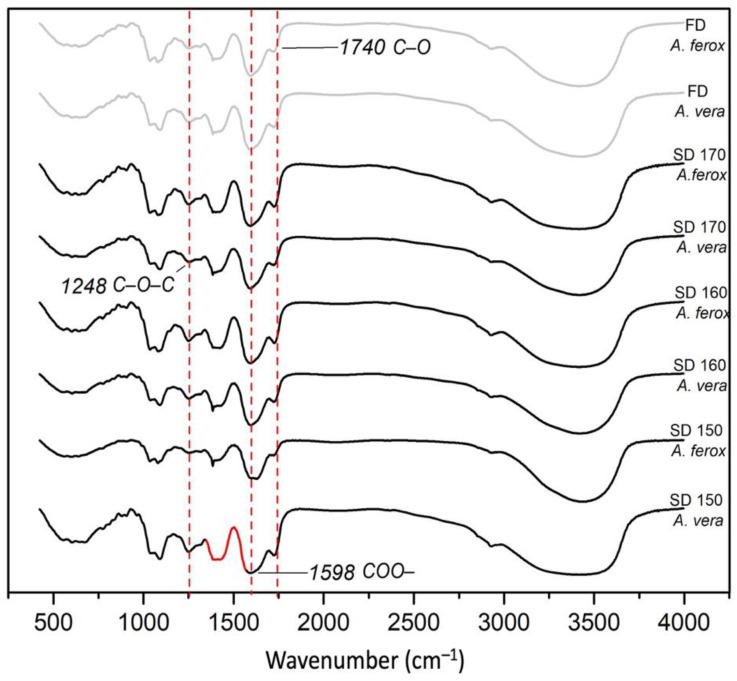
FTIR spectra of *A. ferox* and *A. vera* dehydrated by FD and SD at different temperatures.

The FTIR spectra of the *A. ferox* and *A. vera* dehydrated samples are shown in Figure 3. As can be seen, FTIR profiles from the different SD Aloe samples exhibited the characteristic absorption bands at 3420 cm^−1^ corresponding to –OH groups, 2923 cm^−1^ of the –CH stretching, 1760–1740 cm^−1^ of the C–O stretches of the acetyl group, 1650–1578 cm^−1^ of the C–O, 1598 cm^−1^ of the COO– asymmetric stretching, 1428 cm^−1^ of the CH3 and COO– symmetric stretching, C–O–C stretches of acetyl groups (1248 cm^−1^) [11,41]. Additionally to this, the absorption of ether C–O–C in sugars (1091–1030 cm^−1^) and glucan bands (1031 cm^−1^) were also detected. In *Aloe vera*, the occurrence of the bioactive acetylated polymer acemannan has been associated with the strong bands at around 1740 cm^−1^, 1598 cm^−1^, and 1248 cm^−1^ corresponding to the presence of C–O, COO–, and C–O–C stretches of acetyl groups, respectively [3,11]. It has been reported that acetyl groups play a key role in the interaction of acemannan with other biomolecules, such as phenolic compounds, enhancing their absorption in the intestine [3,42,43]. The vibrational information obtained within the range of 1078–1036 cm^–1^ has been attributed to mannose and glucose, the main monomers comprising the Aloe polysaccharides [41].

**Figure 4:**
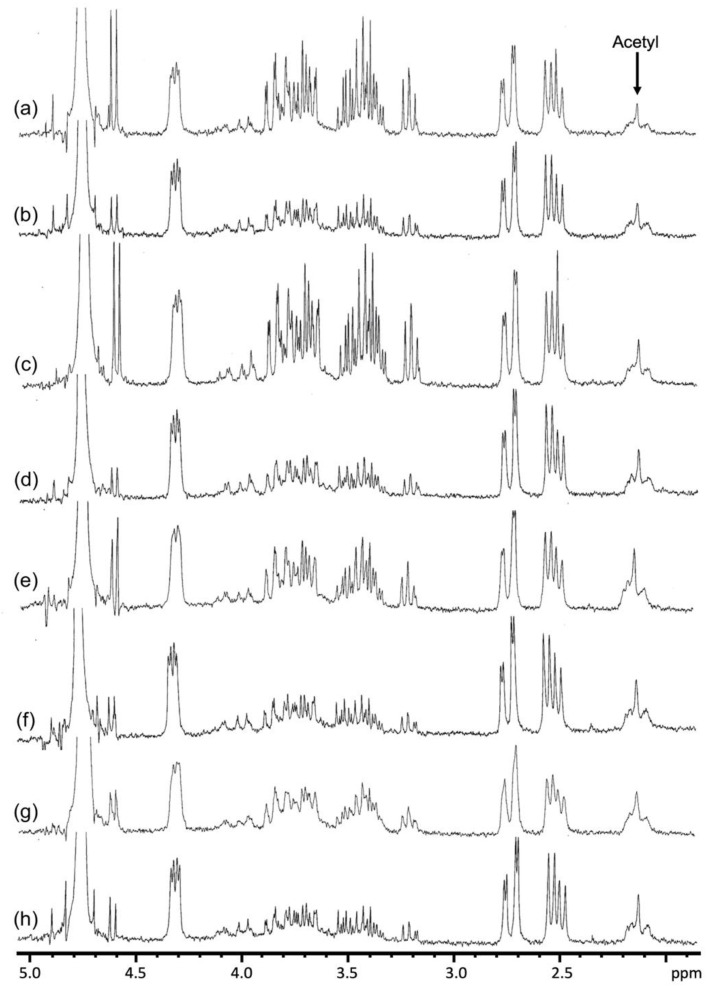
^1^H NMR spectra of the *A. ferox* and *A. vera* dehydrated by FD and SD at different temperatures: (**a**) FD *A. ferox*, (**b**) FD *A. vera*, (**c**) SD 150 *A. ferox*, (**d**) SD 150 *A. vera*, (**e**) SD 160 *A. ferox*, (**f**) SD 160 *A. vera*, (**g**) SD 170 *A. ferox* and (**h**) SD 170 *A. vera*.

^1^H NMR analysis also confirmed the presence of acemannan in all aloe samples, both *A. ferox* and *A. vera*, exhibiting the characteristic signal (2.00–2.26 ppm) corresponding to the acetyl groups present in the mannose units (Figure 4), which have been considered as a fingerprint of this polymer [3,5,39,40,44]. ^1^H NMR analysis revealed the preservation of acetyl groups in all SD samples, accounting for a degree of acetylation higher than 90% in all the SD *A. ferox* mucilages. The preservation of the acetyl groups has been considered an important quality criterion in Aloe-based dehydrated products [3,5] since these groups are involved in most of the beneficial properties associated with Aloe plants, particularly stimulation of the immunological system [3,12,45,46]. It is important to note that these results provide enough scientific evidence about the occurrence of a highly acetylated mannan, similar to the acemannan polymer, in *A. ferox*, making this crop an important alternative in the search for new raw materials for the development of valuable functional ingredients.

^1^H NMR spectra also showed a signal at 4.6 ppm, which has been associated with glucose [39]. Interestingly, this signal was more intense in *A. ferox* samples than in *A. vera*, which could be attributed to the large amount of free glucose in *A. ferox* previously reported by O’Brien et al. [16]. These results suggest that ^1^H NMR could be a useful tool not only to identify or quantify the presence of acetyl groups but also to distinguish *A. ferox* from *A. vera*.

### 3.6. Functional Properties

Functional properties, such as Sw, WRC and FAC, are probably some of the most important technological aspects to be considered for the development of Aloe products since these properties have been associated with certain health benefits, such as hypocholesterolemia and hypoglycemia [21,47]. However, Aloe processing may cause important changes in these techno-functional properties [3,5,11,48]. For this reason, Sw, WRC and FAC of AIRs obtained from *A. ferox* and *A. vera*, dehydrated either by FD or by SD, were determined.

**Figure 5:**
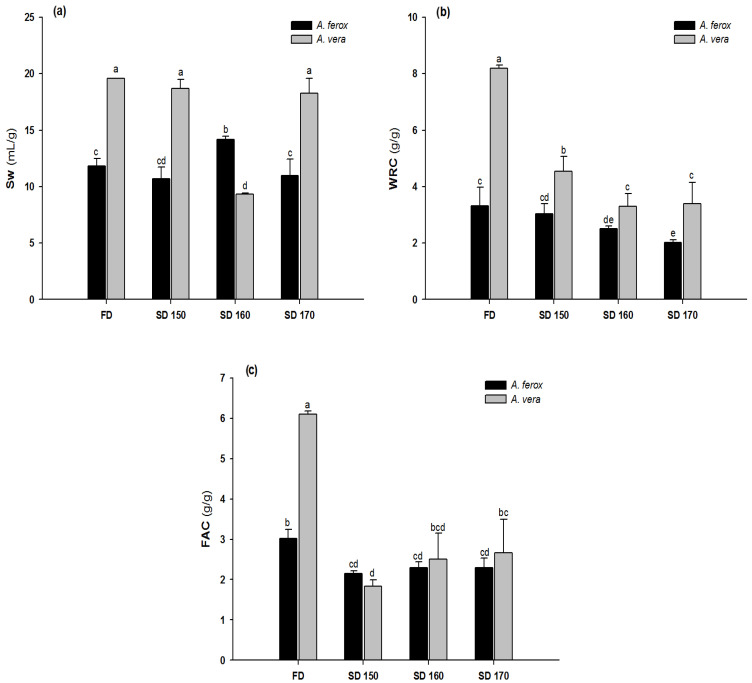
Functional properties of *A. ferox* and *A. vera* dehydrated by FD and SD at different temperatures. (**a**) Sw, (**b**) WRC and (**c**) FAC. Lowercase letters (a, b, c, d, e) above the bars indicate statistical difference in samples (*p* < 0.05).

As can be seen in Figure 5, the functional properties were influenced not only by processing but also by the Aloe variety. Thus, the hydration properties, such as Sw and WRC, of *A. ferox* were significantly lower than those for *A. vera* (*p* < 0.05). Interestingly, no significant differences were observed in the FAC of all SD samples, either for *A. ferox* or *A. vera* (*p* > 0.05).

In particular, the Sw of the SD *A. ferox* ranged from ~10 mL/g, in SD 150, to ~15 mL/g in SD 160. On the contrary, the Sw of *A. vera* ranged from ~9 mL/g in SD 160 up to >18 mL/g in the other samples, including the FD sample (Figure 5a).

In general, the SD process caused a significant decrease in WRC, in particular for *A. vera*, in comparison with the reference samples (Figure 5b). A WRC value of around 3 g/g AIR was measured for the FD *A. ferox* sample, whereas SD samples exhibited lower values (*p* < 0.05). Moreover, the WRC of *A. vera* was drastically reduced, more than 50%, when samples were dehydrated by SD (*p* < 0.05). Therefore, it is clear that the WRC was better preserved in *A. ferox* than in *A. vera*. It has been observed that the decrease of hydration-related properties, and in particular of the WRC, may lead to the loss of some beneficial properties associated with *A. vera*, such as the reduction of blood glucose [47,49,50,51]. The hydration properties of *A. vera* have also been related to the hydrophilic nature of the fully acetylated acemannan, which decreases when deacetylation occurs [3].

The FAC of *A. ferox* decreased from ~3 g/g in the FD sample to ~2 g/g in all SD samples, whereas in the case of *A. vera*, the decrease of FAC values was higher than 66% (SD 150) in comparison with the FD *A. vera* (~6 g/g). Interestingly, an increase in drying temperature did not have a significant effect on FAC, either for *A. ferox* or *A. vera* (see Figure 5c). The reduction of FAC from dehydrated *A. vera* has previously been reported by Femenia et al. [52] and Minjares–Fuentes et al. [5]. In fact, Minjares–Fuentes et al. [5] observed that industrial drying procedures caused a high degradation of the FAC, reaching ~85%. It is important to note that the capacity to reduce the levels of cholesterol, carcinogens and other toxic compounds attributed to the aloe plant has been associated with the ability of aloe polysaccharides to bind organic molecules [6].

From a chemical point of view, changes in the functional properties have been related to the structural and compositional alterations of the acemannan polysaccharide as a consequence of thermal degradation, affecting its three-dimensional structure and the ability to interact with other molecules [3,5,6,52].

## 4. Conclusions

The *Aloe ferox* (*A. ferox*) mucilage dehydrated by SD has been evaluated as a new functional food ingredient. This mucilage presented a similar morphology after being dehydrated by spray drying to that obtained from *A. vera*. However, the SD *A. ferox* mucilage showed better retention of color characteristics, exhibited higher values for functional properties, such as Sw, WRC and FAC and also, an antioxidant capacity better retained than in the case of *A. vera*. Interestingly, from the carbohydrate composition, the presence of a highly acetylated (degree of acetylation > 90%) mannose rich-polysaccharide was inferred, the predominant polymer being in the *A. ferox* mucilage. In general, these features are similar to those of the bioactive polysaccharide acemannan identified in *A. vera*. The degree of acetylation, which was determined through ^1^H NMR and FTIR techniques, is a key aspect since it determines the functionality and the bioactive properties of most mannose-rich polymers.

From this point, we would like to highlight that this is the first study reporting a mannose rich-polysaccharide, with a high degree of acetylation, as the main polymer present in *A. ferox* mucilage, but a more exhaustive structural analysis is required in order to identify the acetylation pattern and the molecular weight of this polysaccharide. On the other hand, these findings could offer new alternatives for the use of *A. ferox* mucilage in the development of new functional Aloe-based food ingredients, although further studies are needed in order to assess the beneficial properties of SD *A. ferox* mucilage for its potential application as a potential new ingredient in many food products.

## Figures and Tables

**Table 1:** *L*, a* and b* color coordinates, *C*, *H*° and Δ*E* values of *A. ferox* and *A. vera* dehydrated by FD and SD at different temperatures.

		*L*	a*	b*	*C*	*H°*	Δ*E*
**FD**							
	*A. ferox*	74.1 ± 0.0 ^e^	−0.6 ± 0.0 ^e^	18.1 ± 0.0 ^abc^	18.1 ± 0.0 ^abc^	91.9 ± 0.9 ^a^	-
	*A. vera*	87.4 ± 0.0 ^a^	−0.8 ± 0.0 ^e^	17.2 ± 0.0 ^bc^	17.2 ± 0.0 ^bc^	92.7 ± 1.1 ^a^	-
**SD 150**							
	*A. ferox*	85.7 ± 2.3 ^ab^	1.4 ± 0.0 ^d^	17.0 ± 2.7 ^bc^	17.1 ± 2.7 ^bc^	85.3 ± 0.8 ^b^	11.7 ± 1.8 ^a^
	*A. vera*	83.1 ± 1.2 ^bc^	1.8 ± 0.3 ^cd^	16.0 ± 0.7 ^c^	16.1 ± 0.7 ^c^	83.4 ± 1.0 ^bc^	5.8 ± 0.9 ^cd^
**SD 160**							
	*A. ferox*	83.4 ± 1.9 ^bc^	2.4 ± 0.8 ^bc^	18.9 ± 1.1 ^ab^	19.1 ± 1.2 ^ab^	82.7 ± 1.8 ^cd^	9.8 ± 0.5 ^b^
	*A. vera*	81.9 ± 2.0 ^c^	2.5 ± 0.5 ^bc^	16.5 ± 0.7 ^c^	16.4 ± 0.7 ^c^	81.4 ± 1.4 ^cd^	7.0 ± 0.9 ^c^
**SD 170**							
	*A. ferox*	77.9 ± 3.4 ^d^	3.1 ± 0.9 ^b^	19.3 ± 2.1 ^ab^	19.6 ± 2.2 ^a^	81.0 ± 1.6 ^d^	5.4 ± 0.4 ^d^
	*A. vera*	77.6 ± 0.8 ^d^	4.2 ± 0.3 ^a^	19.7 ± 0.5 ^a^	20.2 ± 0.6 ^a^	77.9 ± 0.7 ^e^	11.3 ± 0.3 ^a^

Lowercase letters (a, b, c, d, e) indicate statistical difference in samples (*p* < 0.05).

**Table 2:** Carbohydrate composition of *A. ferox* and *A. vera* dehydrated by FD and SD at different temperatures.

Sample		Carbohydrate Composition (mol%)							
		Rha	Fuc	Ara	Xyl	Man	Gal	Glc	UA
**FD**									
	*A. ferox*	2.0 ± 0.0	0.6 ± 0.1	2.1 ± 0.0	1.2 ± 0.0	59.5 ± 5.1	3.7 ± 0.1	20.8 ± 0.4	10.0 ± 0.4
	*A. vera*	1.2 ± 0.0	0.2 ± 0.0	2.0 ± 0.0	0.8 ± 0.1	69.7 ± 3.6	2.9 ± 0.0	14.8 ± 0.3	8.4 ± 0.2
**SD 150**									
	*A. ferox*	0.0 ± 0.0	0.0 ± 0.0	2.2 ± 0.0	0.0 ± 0.0	76.3 ± 2.6	3.1 ± 0.0	7.8 ± 0.3	10.6 ± 0.3
	*A. vera*	1.2 ± 0.0	0.4 ± 0.0	1.7 ± 0.1	0.6 ± 0.0	76.8 ± 4.5	2.7 ± 0.0	8.7 ± 0.3	7.9 ± 0.0
**SD 160**									
	*A. ferox*	0.0 ± 0.0	0.0 ± 0.0	1.4 ± 0.0	0.0 ± 0.0	72.7 ± 2.3	1.5 ± 0.0	7.5 ± 0.0	16.9 ± 0.4
	*A. vera*	0.5 ± 0.0	0.0 ± 0.0	1.9 ± 0.0	0.0 ± 0.0	76.4 ± 0.3	2.5 ± 0.1	9.4 ± 0.0	9.2 ± 0.1
**SD 170**									
	*A. ferox*	0.4 ± 0.0	0.0 ± 0.0	1.5 ± 0.0	0.6 ± 0.0	74.7 ± 5.3	2.3 ± 0.1	8.0 ± 0.3	12.5 ± 0.2
	*A. vera*	1.1 ± 0.0	0.0 ± 0.0	1.7 ± 0.0	0.0 ± 0.0	74.8 ± 3.7	3.1 ± 0.0	10.3 ± 0.2	9.0 ± 0.1

## Data Availability

Data is contained within the article.
